# Revisiting Conservative Approaches to Fracture Care Such As Close Contact Casting in the Elderly Co-morbid Patient: A Technical Report

**DOI:** 10.7759/cureus.98907

**Published:** 2025-12-10

**Authors:** Stephen R Alfred, Anthony J Thayaparan

**Affiliations:** 1 Medicine, Royal Surrey County Hospital, London, GBR; 2 Trauma and Orthopaedics, Imperial College Healthcare NHS Trust, London, GBR; 3 Medicine, Cardiff University, Cardiff, GBR

**Keywords:** ankle fracture, close contact cast, conservative fracture management, distal radius fracture, risk vs benefit assessment

## Abstract

Close contact casting (CCC) is a non-surgical treatment for certain types of fractures, using tight moulding to achieve anatomical alignment. During the COVID-19 pandemic, when healthcare systems globally were under severe strain, conservative management such as CCC was a more widely used alternative to surgical management. However, it has since been considered that a large cohort of patients over 60 may have similar functional outcomes, fewer complications, and lower associated costs when comparing CCC to surgical management. Currently, there is no validated tool to determine which patients could be more suitable for CCC. This technical report proposes the surgical risk vs benefit assessment of fracture management in the elderly (SAFME) scoring tool. The SAFME scoring tool uses thirteen parameters of patient demographics to objectively evaluate conservative management against surgery, and guide clinicians’ decision-making for treatment. Furthermore, a fracture clinic protocol (FCP) being incorporated into practice would allow for the careful multidisciplinary monitoring of elderly patients with fractures who have followed the CCC pathway, ensuring safe and effective care. The SAFME scoring tool would require testing through retrospective analysis and prospective trials to refine scoring thresholds, evaluate safety, and assess its cost-effectiveness before implementation. If validated, the scoring tool could be used to support more individualised, evidence-based, and resource-efficient fracture management in elderly adults.

## Introduction

The emergence of the COVID-19 pandemic in 2020 increased the burden on healthcare systems around the world [1]. The significant rise in patient numbers caused several issues that led to strain in multiple facilities. This created a knock-on effect on other specialities. Unwell patients requiring hospitalisation reduced the number of inpatient beds for those who required operative management under orthopaedics as well as other surgical specialities [2]. While emergency surgical care must continue through times of high demand on healthcare systems, those that are deemed urgent or should be expedited (perhaps requiring surgical management within two weeks of onset of injury, such as in ankle fractures) may not occur so readily due to delay in prioritisation of more urgent or emergency operations and limited theatre facilities. A study by Khroud-Dhillon et al. [3] showed that 28% of ankle fracture cases admitted to Lister Hospital during March to June 2020, at the height of the pandemic, were treated operatively, compared to 80% the previous year in the same four-month period. In the same period in 2020, 72% of fractures were treated conservatively compared to 20% in the same period the previous year. Interestingly, this study showed that functional outcomes in conservatively managed patients were non-inferior compared to the conservatively managed patients in the pre-pandemic cohort. To assist clinicians with managing the unprecedented time, the British Orthopaedic Association published COVID BOAST guidelines [4], including stating that "where possible, use non-operative treatment and removable splints, recognising that some may require later reconstruction". Although practices were initially adapted due to the unprecedented pressures experienced during the pandemic, it has been considered whether these practices can be more widely implemented. Although Covid-19 is no longer a serious burden, there is always a need to consider treatments with no significant difference or improved outcomes but associated with reduced complications and lower healthcare costs.

Treatment decisions should balance the benefits of surgery for otherwise healthy patients against the higher risks in those with significant co-morbidities. Patients who are otherwise healthy are at lower risk of post-operative complications, and surgery would allow for early rehabilitation and quicker return to function. However, this must be weighed against risks faced by other patients, particularly those over sixty-five, who are often more comorbid [5]. These patients often have a lower physical functional baseline [6], and surgery may carry more risks [7].

Close contact casting (CCC) is a conservative method used in orthopaedics that helps provide anatomical alignment to certain fractures, such as unstable ankle fractures. While a stable fracture is a break in a bone where the bone fragments can remain in correct anatomical alignment under normal physiological stresses without a tendency to displace, an unstable fracture is one in which a broken bone cannot remain in position without an intervention, such as surgery or external support. This can be due to the nature of the fracture or damage to structures that act as stabilisation. If the broken bone cannot remain in position, it will not be able to heal properly. This can cause pain, deformity, and loss of function. Therefore, fixation is required, which can be internal or external. Internal fixation is the use of screws, plates, or rods in surgery to provide stability to the bone, whereas CCC provides non-surgical external fixation. In CCC, an externally padded cast is applied to a limb by a surgeon, using moulded pressure to ensure alignment. A standard cast is not an option in unstable fractures as it cannot control or resist forces that make bone fragments move out of place due to its loose and uneven fitting. CCC has been used to provide an alternative to surgery, especially in elderly patients [8], but until now its use has been limited. This technical report proposes that there is a large cohort of adults over 60 who could be treated conservatively with CCC rather than surgical intervention.

The Ankle Injury Management (AIM) trial by Willett et al. [9] was a randomised-controlled trial in adults over 60 with a mean age of 71 years, who were recruited and placed into either a group of CCC or operative management. Patients belonging to the CCC group had undergone manipulation under general or spinal anaesthesia, followed by application of a total-contact cast. After treatment was complete, the initial study was followed up for six months, showing comparable Olerud-Molander Ankle Scores (OMAS) of 66.0 and 64.5 for surgery and CCC, respectively (where the higher the number from 0-100, indicated better the outcomes and fewer symptoms). The rates of malunion and medial malleolar non-union were higher in those treated with CCC (15% and 7% respectively). Patients undergoing operative fixation, however, had a 3% malunion rate and 1% medial malleolar non-union. Those with these two outcomes were noted to have a lower OMAS. The data show that malunion and non-union rates were higher in CCC, but overall, CCC and surgical management had similar OMAS. There are several reasons for this. Patients in the CCC group healed well and reported good function, with malunion and non-unions only occurring in a minority of patients. Patients who were surgically managed still experienced complications such as infections, wound issues, and re-operations, which reduced their OMAS. However, it must be considered that this was their OMAS at six months. Malunion and non-union can cause pain and stiffness that occur 1-2 years later. It must also be considered that small degrees of angulation or rotation causing poor union of bone may not impair walking or daily activity in elderly patients, who were the primary participants in this study. The elderly usually have lower functional expectations and activity levels, and the OMAS is used to measure functional recovery after an ankle fracture using nine functional parameters. Therefore, in the elderly, their perceived functional outcome can remain good even with mild malunion.

Keene et al. [10] from the same team economically evaluated the treatment arms of the aforementioned study and found that, on average, £644 was saved by using casts in comparison to surgical management.

Important considerations include intra-operative variability in the method of fixation and differences in implants, but also post-operative care in weight-bearing status and splintage. The aim had been for most participants to partially or fully weight bear four weeks after intervention. The CCC group was treated with a more consistent post-procedural plan, usually allowing early mobilisation, which promotes confidence, muscle strength, and circulation. For those who had surgical fixation, the post-operative management, including progression of weight-bearing status, was ultimately at the discretion of the operating surgeon. Weight bearing through a limb is considered ideal to improve blood flow and prevent stasis, but may not be possible in some cases. For example, a patient with poor mobility who then experiences a distal radius fracture may have an intervention performed and be discharged with appropriate venous thromboembolism (VTE) prophylaxis, to aid nursing needs and personal care provision.

Keene et al. [11] performed a further study of a three-year follow-up of those in the AIM trial, further confirming the comparability of CCC to surgical fixation. Surgically treated patients had a mean OMAS score of 79.4 at three years, while those who underwent CCC similarly had a score of 76.3. One must consider that, while the usage of CCC had been shown to be cost-effective and reduced potential post-operative complications, patients were also undergoing anaesthetic procedures.

More recently, the trial by Carter et al. [12] demonstrated that in the management of unstable medial malleolus fractures, patients who underwent surgical fixation had similar OMAS to non-surgical fixation after one year. The study did note that 13 patients in the conservatively managed group had significantly higher rates of radiological non-union (20%); however, only one required further surgery. Longer-term follow-up is required to determine any future implications. Importantly, the study also noted that the fracture type and reduction quality should be radiographically assessed before deciding treatment option. Overall, the results would indicate that selective non-fixation of anatomically reduced medial malleolar fractures is an acceptable treatment method with similar functional outcomes.

It is important to consider that there are also recent papers that favour operative fixation. Toru et al. [13] compared operative vs non-operative management of isolated Weber B ankle fractures, which showed significantly higher OMAS in the operative group. However, the authors of the paper attribute the lower OMAS in the non-operative group to prolonged immobilisation and fears of residual deformities. A significant proportion (74%) of conservatively managed patients stated satisfactory functional recovery, giving further credence to this management option. It should be noted that OMAS is a patient-reported functional outcome score that was measured at six months in this study. Arthritis and other complications may take longer to set in and require long-term follow-up to determine patient satisfaction.

Systematic reviews and meta-analyses were also included in the search criteria for studies comparing conservative and operative management of ankle fractures.

Elgayar et al. [14] compared surgical and conservative treatment in unstable ankle fractures in adults. Only studies with a minimum of six months follow-up were included, with only five randomly controlled trials (RCTs) meeting the inclusion criteria. It should also be noted that different ankle fracture classification systems were used, as there is no unified classification system. Although the oldest studies, Baeuer et al. [15] and Phillips et al. [16] had the longest follow-ups, at seven and 3.5 years respectively, both reported no significant difference in clinical outcomes. Importantly, these two studies reported radiological signs of osteoarthritis as being higher in the conservatively managed group. However, these trials did not use a validated functional outcome measure. Of the studies that did use OMAS, Makwana et al. [17] reported a better score for the surgical group at 27 months. Sanders et al. [18] and Willet et al. [9] found no significant difference at six and 12 months, respectively. Only these three studies included important demographic details such as regular substance use, alcohol and smoking history, as well as past medical history. Therefore, it should be noted that across the five studies included, there was a lack of detailed information regarding patient demographics, although these factors are known to impact management outcomes.

Tian et al. [19] conducted a systematic review and meta-analysis comparing operatively and non-operatively treated isolated Weber B ankle fractures. This study showed that operative and non-operative management produced no significant differences in OMAS or Foot and Ankle Outcomes Questionnaire (FAOM) scores; however, it also noted a significantly higher American Orthopaedic Foot and Ankle Score (AOFAS) as well as a lower visual analogue scale (VAS) in non-surgically managed patients. The AOFAS score combines pain, function, and alignment scores for a total score that indicates how satisfied the patient is with the outcome of their treatment. VAS is a scale on which patients can indicate their level of pain. One reason these produced more favourable scores in conservatively managed patients may be that surgery can often cause more pain that can be longer lasting, which is not the case in conservatively managed patients. Peng et al. [20] conducted a meta-analysis comparing surgical and conservative management of adult ankle fractures. This study did note higher OMAS in surgically managed patients at six months and after 24 months. However, another questionnaire, the short form-12 (SF-12), demonstrated that the psychological impact of patients receiving surgery after 12 months was significantly worse than those conservatively managed. A possible reason for this is that patients may not have been informed of the pain involved in surgical fixation and the limitations the pain causes to their function, compared to conservative management.

Another significant limitation in directly comparing study outcomes is heterogeneity in fracture classification across the trials. Several major studies, such as the AIM trial, used pragmatic descriptors such as unstable, without using a consistent radiological definition or formal classification framework. Earlier studies used the Weber ankle fracture classification system. The lower leg consists of two bones, the tibia and fibula, and in combination with the talus (a bone in the foot), they form the ankle joint. The ankle joint is also stabilised by other structures such as ligaments and muscles. The syndesmosis is an example of a ligament that attaches the tibia and fibula at the ankle. The Danis-Weber classification system classifies fractures according to where the fracture is in relation to the syndesmosis. More recent studies have used Lauge-Hansen patterns or isolated descriptors of the injury, such as the presence of talur shift. The Lauge-Hansen classification system is based on a combination of foot position and direction of force applied at the time of injury. Clearly, these systems capture different anatomical and biomechanical dimensions of stability. It should also be noted that two different injuries could be classified similarly in one system but differently in another.

The inconsistency in the classification of fracture patterns limits the comparability of operative and non-operative cohorts across studies. For example, some trials focused on Weber B ankle fractures, which it could be argued are inherently more stable and so can report more acceptable outcomes when conservatively managed. Conservative management may seem more effective in studies with milder fracture patterns, or conversely, inferior in studies with more unstable patterns, even though underlying patient populations differ. The variations cause clinical heterogeneity, which could partly explain conflicting conclusions across the literature, complicating meta-analysis.

Javed et al. [21] conducted a systematic review and meta-analysis for surgical vs conservative management of ankle fractures in adults. Data from the studies by Willett et al. [9] and Keene et al. [11] were included. Different functional outcome tools were used to measure results, such as OMAS and FAOM. Overall, there was no statistically significant difference between surgical and conservative groups at six months or long-term follow-up in four of the six studies. Of the other two, the study by Makwana et al. [17] favoured surgery, but according to the Good Research for Comparative Effectiveness (GRACE) quality assessment, it was at high risk of bias, and the study by Mittal et al. [22] favoured conservative management. Mittal et al. [22] was a study of AO-type-B1 fractures with minimal talur shift, which showed better ankle function scores in conservatively managed patients. Conservatively managed patients did have higher levels of early treatment failure, but it should be considered that this might have been deemed acceptable to avoid surgery.

This is particularly integral in those who suffer long-term medical co-morbidities, but with unstable injuries such as an ankle fracture with syndesmotic injury in a 70-year-old patient with a past medical history involving diabetes mellitus and kidney disease. Subsequent potential infection with a respiratory illness in this at-risk group of people could be an unnecessarily life-threatening complication.

In the context of the above patient with the unstable ankle fracture, there are, of course, other risks associated with surgery, such as implant or fixation failure and wound complications. Conservative approaches such as CCC should be considered in these patients if they are able to comply with post-procedural instructions, and this can be performed safely.

A potential solution to the above dilemma would be for patients who presented to their local emergency department at the time of injury to receive adequate reduction by a senior team aiming for appropriate anatomical alignment. The patient would then be sent home for a period to allow for reduction in swelling and pain, with appropriate VTE prophylaxis and safety clearance for mobilisation with aids. It is normal practice in the United Kingdom that patients are subsequently called into their local fracture clinic to check the level of swelling around the ankle fracture prior to operative fixation.

This technical report proposes an expansion of the current fracture clinic service through a Fracture Clinic Protocol (FCP). This would be incorporated into the current fracture clinic practice, incorporating more senior clinicians performing CCC for those having a higher probability of post-operative complications, but able to comply with weight-bearing instructions and sufficiently well enough to remain an outpatient. This would ensure comfort and safety at home while reducing associated surgical risks and restoring function.

One may question the need for expansion of the current fracture clinic service requiring specialist expertise and resources, with only a comparatively modest cost-saving as discussed above. However, aside from ever-evolving healthcare pressures, the general trend of fracture admissions was shown to be increasing by Jennison and Brinsden [23], suggesting a longer-term benefit of the expansion of the current fracture clinic service. Virtual fracture clinics have been an increasing presence, typically used for lower-risk, stable injuries, and providing remote management. The concept of FCP would be to consider patients who are in the high-risk cohort, where surgery is undesirable, requiring input from senior orthopaedic doctors for their knowledge, experience, and specifically to assist with the hands-on procedure that is CCC.

The British Orthopaedic Foot and Ankle Society (BOFAS) certainly gives some credence to this proposal, as evidenced in the BOFAS hyperbook [24]. They state that if there is any concern regarding the stability of a conservatively managed ankle fracture, the patient should be reviewed face-to-face in clinic in 7-10 days, with a standing X-ray on arrival. BOFAS goes on to state that if potentially unstable fractures are treated non-operatively, it may be necessary to follow the patient weekly with serial X-rays to assess the position of the fracture, with cast removal at six weeks.

It has been noted that there is variable practice in the UK despite BOFAS advice, and it is more common practice to use selective rather than weekly radiographs unless earlier indicated. However, this is because most injuries treated in this pathway are currently stable.

Studies that have been done show that currently, BOAST guidelines regarding ankle fracture management are not being adhered to. The Acute Management of Ankle Fractures (AUGMENT) study by Fennelly et al. [25] was done to check compliance of ankle fracture management with BOAST 12 guidelines. It demonstrated that only a quarter of conservatively managed patients in the study received follow-up in six weeks. Of the patients not followed up in this time period, two-thirds were Weber A injuries who were being treated with a walking boot. Another study by Gokhale et al. [26] showed that only 22% of patients in the study who were treated non-operatively had a weight-bearing X-ray. This then caused a delay in early weight bearing and worsened patient outcomes. The aim of the development of FCP would allow for the advice and guidelines by BOFAS and BOAST to be adhered to. Morcos et al. [27] performed a closed-loop audit to determine if BOAST guidelines regarding weight-bearing X-rays being performed within two weeks of injury, where there is uncertainty of stability in a fracture pattern. The intervention was a stability-based management protocol that incorporated local teaching. The closed-loop audit demonstrated a significant increase in performing stability X-rays in fracture clinic follow-up from 70% to 85.7%. This provides evidence that implementation of a protocol will improve compliance.

The Fractured Ankle Management Evaluation (FAME) trial, currently in progress based at the University of Oxford, aims to push the boundaries further, suggesting the proposed method of CCC be used in those under the age of 60, and as young as 18 [28]. While there may be benefits to not requiring surgery and risk of anaesthesia and operative risks as mentioned above, in this likely fitter, healthier, and more mobile cohort, one may consider that operative management enables faster return to function. The elderly population may be generally deemed to perform lower-demand activities (typically referred to as aged over 60 in this article); their return to function may be slower. This also takes into consideration the social background, where the younger individual is more likely to be of working age and thus may require a return to normal activity sooner. With more favourable physiology in the 18-60 years age group, healing is likely to be faster and therefore subjecting patients to CCC for even potentially four weeks may be deemed unnecessary and potentially risk them with effects associated with prolonged immobility (such as VTE).

Often, patients with successful initial manipulations of fractures treated in devices such as plaster of Paris casts are monitored subsequently for a period, especially when loss of reduction is higher (usually within the first two weeks). Often, it is the case that a further loss of reduction may suggest that the injury has been unsuccessfully managed conservatively, and, therefore, operative management may be indicated for definitive fixation. Again, in such situations where the health service is under great strain, it may be reasonable to consider safe re-manipulation under adequate analgesia.

The results of the Distal Radius Acute Fracture Fixation Trial 2 (DRAFFT 2) by Achten et al. [29] will certainly be interesting to observe and indeed provide potential evidence supporting the above idea, where percutaneous fixation of dorsally displaced distal radius fractures is being compared to cast-immobilised fractures of the same nature. The influential first study by Costa et al. [30] showing comparative functional outcomes of volar locking plates and percutaneous wire fixation of distal radius fractures certainly gives some credence to this. Employing the principles of CCC early and appropriately (such as after the reduction of significant swelling) may provide further stability compared to conventional plaster of Paris applications in the acute setting, with a potential loss of reduction after completion of the same cast.

Current guidelines regarding ankle fractures are limited. The National Institute for Health and Care Excellence (NICE) has guidelines [31] for the non-surgical management of unimalleolar ankle fractures and states that if treating ankle fractures with surgery, consider operating on the day or the day after. Beyond this, NICE guidelines for ankle fracture management are limited, demonstrating a research gap. BOAST also offers some guidance on managing ankle fractures, regarding patients over 60 [8]. It states that in patients over 60, CCC is an option if reduction can be maintained. This gives credence to managing ankle fractures with CCC, but it must be considered if it is the best option and what makes it so. There is currently no validated tool to objectively guide the selection of elderly patients for whom conservative management may be most beneficial. The purpose of this technical report is to propose such a framework using a novel scoring tool in combination with the Fracture Clinic Protocol (FCP) that would also allow for the careful monitoring of this cohort.

## Technical report

The proposal for the Surgical Risk vs Benefit Assessment of Fracture Management in the Elderly (SAFME) scoring tool has been made because of increasing evidence that CCC produces similar outcomes to surgery. To highlight this, an evidence scan of literature was conducted to identify studies comparing conservative and surgical management of unstable ankle fractures in adults. The purpose was not to conduct a full systematic review but to follow a structured format to identify relevant studies in this area, with greater emphasis on more recent studies. Searches were performed in MEDLINE and Cochrane Library, using a combination of the terms: ankle fracture, unstable, surgical, conservative, non-operative, and close contact cast. Reference lists of relevant systematic reviews and meta-analyses were also screened to identify additional relevant trials. Inclusion criteria were: randomised or prospective studies, adults with displaced or unstable ankle/ distal radius fractures, studies comparing surgical and cast-based management, with all studies including at least a six-month follow-up. The selected studies are summarised in the table below. This structured synthesis aims to provide a clearer understanding of all the evidence base underpinning the rationale for considering CCC in elderly patients with ankle fractures.

**Table 1:** Summary table of studies comparing surgery and conservative management of unstable ankle fractures AIM Trial - The Ankle Injury Management Trial; RCT - randomized control trial; CCC - close contact casting; ORIF - open surgical reduction and internal fixation; OMAS - Olerud-Molander Ankle Score; QALY - auality-adjusted life year; FAOM - Foot and Ankle Outcomes Questionnaire; AOFAS - American Orthopaedic Foot and Ankle Score; VAS - visual analogue scale; SF-12 - short form-12; OA - osteoarthritis

Study	Design/ sample size	Patient population	Fracture types	Compared interventions	Key outcomes	Conclusions
Willett et al., 2014 [9] (AIM Trial)	RCT; n=620	Adults >60 (mean 71)	Unstable ankle fractures	CCC with manipulation under anaesthesia vs ORIF	Six-month OMAS: 64.5 (surgery) vs 66.0 (CCC). Higher malunion/non-union in CCC (15%/7%) vs surgery (3%/1%). The surgical group had more wound complications.	No significant difference in functional outcome.
Keene et al., 2016 [10](economic evaluation)	Health-economic analysis (AIM trial data)	Same as above	Same as above	CCC vs ORIF	CCC saved ~£644 per patient; similar QALYs.	CCC is cost-effective compared with surgery.
Keene et al., 2018 [11] (AIM three-year follow-up)	Prospective RCT follow-up	Adults >60	Unstable fractures	CCC vs ORIF	Three-year OMAS: 76.3 (CCC) vs 79.4 (surgery); no significant difference.	Long-term functional outcomes remain comparable.
Carter et al., 2024 [12]	RCT; n=154	Adults with unstable medial malleolus fractures	Isolated medial malleolus (after fibular stabilisation adequacy)	Conservative vs operative	One-year OMAS was similar between groups; 20% radiological non-union in conservative management (only one required surgery). Radiographic assessment was essential.	Selective conservative management of well-reduced fractures is safe with comparable outcomes.
Toru et al., 2023 [13]	Retrospective cohort study, n=115	Adults with isolated Weber B fractures	Weber B	Conservative vs operative	Higher OMAS in the surgical group at six months; 74% of conservative patients were still satisfied. Conservative management immobilisation was prolonged.	Surgery gives higher early OMAS but high satisfaction with conservative care; long-term outcomes are unclear.
Elgayar et al., 2017 [14]	Systematic review of five RCTs; n=951	Adults with ankle fractures	Closed ankle fractures, including unstable patterns	Surgical fixation vs conservative (casting)	Review of five RCTs with ≥6-month follow-up	Concluded current evidence is insufficient to definitively support surgery over conservative treatment; highlights the need for more high-quality data.
Tian et al., 2022 [19]	Systematic review and meta-analysis (Weber B)	Adults	Isolated Weber B fractures	Conservative vs operative	No significant difference in OMAS or FAOM. Conservative patients had higher AOFAS and lower VAS pain scores.	Overall, similar functional outcomes; some patient-reported outcomes favour non-operative care.
Peng et al., 2023 [20]	Systematic review and meta-analysis	Adults	Mixed ankle fractures	Conservative vs operative	Surgery was associated with higher OMAS at six and 24 months. SF-12 mental health was worse after surgery at 12 months.	Functional gains with surgery were modest; the psychological impact of surgery was significant.
Javed et al., 2020 [21]	Meta-analysis of seven trials; total n=1153	Adults with displaced or unstable ankle fractures	Various closed ankle fractures (displaced/unstable)	Operative fixation vs conservative management	Pooled functional outcomes: no statistically significant difference at six months (MD≈1.0; CI: 2.3-4.3) or ≥12 months (MD≈4.6; CI: 1.0-10.2). The conservative group had higher malunion/non-union and early failure; the surgical group had a higher risk of reoperation and infection.	Suggests that for many displaced/unstable fractures, non-operative management may achieve similar short-term function but carries a different risk profile.
Makwana et al., 2001 [17]	RCT; n=47	Adults	Weber B unstable fractures	Plaster cast vs ORIF	The surgical group had a better functional outcome at 27 months; the conservative group had more malalignment.	Favoured surgery.
Bauer et al., 1985 [15]	RCT; n=75	Adults	Mixed ankle fracture patterns	Cast vs ORIF	No functional difference at seven years; radiographic OA was more frequent after casting.	No outcome difference.
Phillips et al., 1985 [16]	RCT; n=64	Adults	Mixed ankle fracture patterns	Cast vs ORIF	Similar functional results; OA was more common in the cast group.	No significant difference in clinical outcomes.
Sanders et al., 2012 [18]	RCT; n=42	Adults	Unstable ankle fractures	Conservative vs operative	No functional superiority of either method at 12 months.	No significant difference.
Mittal et al., 2017 [22]	Prospective trial	Adults with AO-B1 fractures (minimal talar shift)	Minimally undisplaced ankle fracture	Cast vs surgery	The conservative group had better functional outcomes, but higher early treatment failure.	Conservative management was reasonable for selected B1 fractures.

The review of the current evidence demonstrated that CCC is a viable alternative to surgical management of certain fractures. However, it was also clear that there is no existing validated tool to guide orthopaedic teams on when CCC may be the preferred treatment option. Using such a scoring tool would standardise decision-making regarding management of certain fractures, whilst clearly highlighting if the advantages of surgical outcome outweigh the associated risks. 

Surgical Risk vs Benefit Assessment of Fracture Management in the Elderly (SAFME) scoring tool

While surgery can offer advantages such as anatomical reduction and early mobilisation, there are several factors that make it potentially riskier for the elderly. Elderly patients often present with multiple co-morbidities, which can significantly increase the risks associated with surgery and anaesthesia [7]. Common conditions in the elderly include cardiovascular diseases, which increase the risk of perioperative cardiac events [32], as well as diabetes, which is associated with higher rates of surgical site infections and poor wound healing [33].

It is generally considered that, due to aging, elderly patients have reduced physiological reserve across multiple organ systems [34]. This results in a diminished capacity to respond to surgical stress, which can lead to a multitude of post-operative complications. Studies demonstrate that post-operative delirium can affect up to 50% of elderly surgical patients [35]. This can lead to prolonged recovery times and extended hospital stays, in turn increasing the risk of hospital-acquired complications [36]. 

Aging also has a significant impact on the musculoskeletal system. Older adults are known to suffer more from osteoporosis and poor bone quality [37]. This has serious implications in surgical fixation as reduced bone density increases the risk of implant failure and loss of fixation [38]. As a result, osteoporotic bone may require more extensive hardware, potentially increasing surgical trauma [39].

It is also vital to consider the broader psychosocial impact of surgical interventions. Studies have demonstrated that longer hospital stays and higher complication rates in elderly surgical patients can significantly increase healthcare costs [40]. Elderly patients who have had surgical interventions that have required a prolonged recovery period may experience loss of independence and reduced quality of life [41], potentially necessitating long-term care arrangements.

Considering these factors, conservative management options like CCC may offer a more favourable risk-benefit profile for certain fractures in the elderly. They can provide adequate fracture stabilization while avoiding the risks associated with surgery and anaesthesia.

To help clinicians determine which elderly patients may be more suitable for CCC rather than surgical intervention, this technical report proposes the Surgical Risk vs Benefit Assessment of Fracture Management in the Elderly (SAFME) scoring tool. This can then be used in practice with a Fracture Clinic Protocol (FCP), allowing careful monitoring of patients for whom it has been determined that CCC is the optimal management. 

The aim would be that this scoring system incorporates early involvement of physiotherapists and occupational therapists in patient care to assist clinicians in forming an accurate understanding of the patient's health status. As our understanding of geriatric fracture care evolves, there's a growing emphasis on comprehensive geriatric assessment and multidisciplinary approaches to optimise outcomes in this vulnerable population. Whereas currently, orthopaedic doctors rely on their knowledge and experience as to which patients may benefit most from surgical or non-surgical management, an objective scoring system would be used to help guide their decision-making more clearly, incorporating factors that are already considered during their decision-making process. Unlike frailty indices, the SAFME scoring tool integrates fracture complexity, anaesthetic risk, functional impact, cognitive ability, and social determinants. 

This scoring system will assess various factors on a scale of 0-3, with higher scores indicating CCC may be more beneficial. The total score will help guide decision-making. The following Table 1 outlines the SAFME scoring tool, including each patient demographic and the associated scoring threshold. 

**Table 2:** Surgical Risk vs Benefit Assessment of Fracture Management in the Elderly (SAFME) scoring tool *based on albumin levels or a validated nutritional assessment tool. **VTE risk assessment according to local guidelines. VTE - venous thromboembolism; ADL - activities of daily living; ASA - American Society of Anesthesiologists

SAFME	0	1	2	3	References
Age	<60 years	61-70 years	71-80 years	>80 years	[7]
Mobility status	Mobilises long distances unaided	Can mobilise >100m with walking aid	Mobilises <100m with walking aid	Primarily chairbound/ bedbound	[42]
Functional status (based on activities of daily living)	Independent	Needs assistance with 1-2 ADLs	Needs assistance with 3-4 ADLs	Dependent for most ADLs	[43]
Weight-bearing status	Fully weight-bearing (FWB)/ adhering to instructions	FWB but variable adherence	Partial weight bearing	Non-weight-bearing/ difficult adherence	[44]
Cognitive status	No cognitive impairment	Mild cognitive impairment	Moderate dementia	Severe dementia	[43]
Bone quality	Normal bone density	Osteopenia	Moderate osteoporosis	Severe osteoporosis	[38,39]
Fracture complexity	Comminuted or complex fracture pattern	Displaced fracture, simple pattern	Minimally displaced fracture	Simple, non-displaced fracture	[45]
Anaesthesia risk	ASA I	ASA II	ASA III	ASA IV or higher	[46]
Polypharmacy	0-2 medications	3-5 medications	6-8 medications	9+ medications	[47]
Nutritional status*	Well-nourished	At risk of malnutrition	Moderate malnutrition	Severe malnutrition	[48]
Skin status in fractured limb	No signs of skin breakdown	Early signs of skin pressure damage	Sore/ blister present	Widespread pressure damage to skin/ deep pressure sores present	[49]
VTE risk	Very low	Low	Medium	High	**
Social support	Strong support system, caregiver available	Moderate support, part-time assistance available	Limited support, lives alone	No support system	[50]

Scoring interpretation is as follows, with a total score range of 0-39:

0-8: lower probability of benefit from conservative management - surgical management may be preferred if indicated by fracture type;

9-24: moderate probability of benefit from conservative management - carefully weigh risks and benefits, consider conservative management;

25-39: higher probability of benefit from conservative management, e.g., CCC - this may be preferred unless surgery is necessary. 

Following assignment of a SAFME score and after discussion with the patient regarding personal preferences, this technical report has devised a protocol that will allow for the continued safe care of patients being managed with CCC. 

Fracture Clinic Protocol

This technical report further proposes the idea of the Fracture Clinic Protocol (FCP) that may be incorporated into current fracture clinic practices that run weekly in trauma and orthopaedic departments. One may consider the following process diagram for a patient's journey through the FCP.

**Figure 1:**
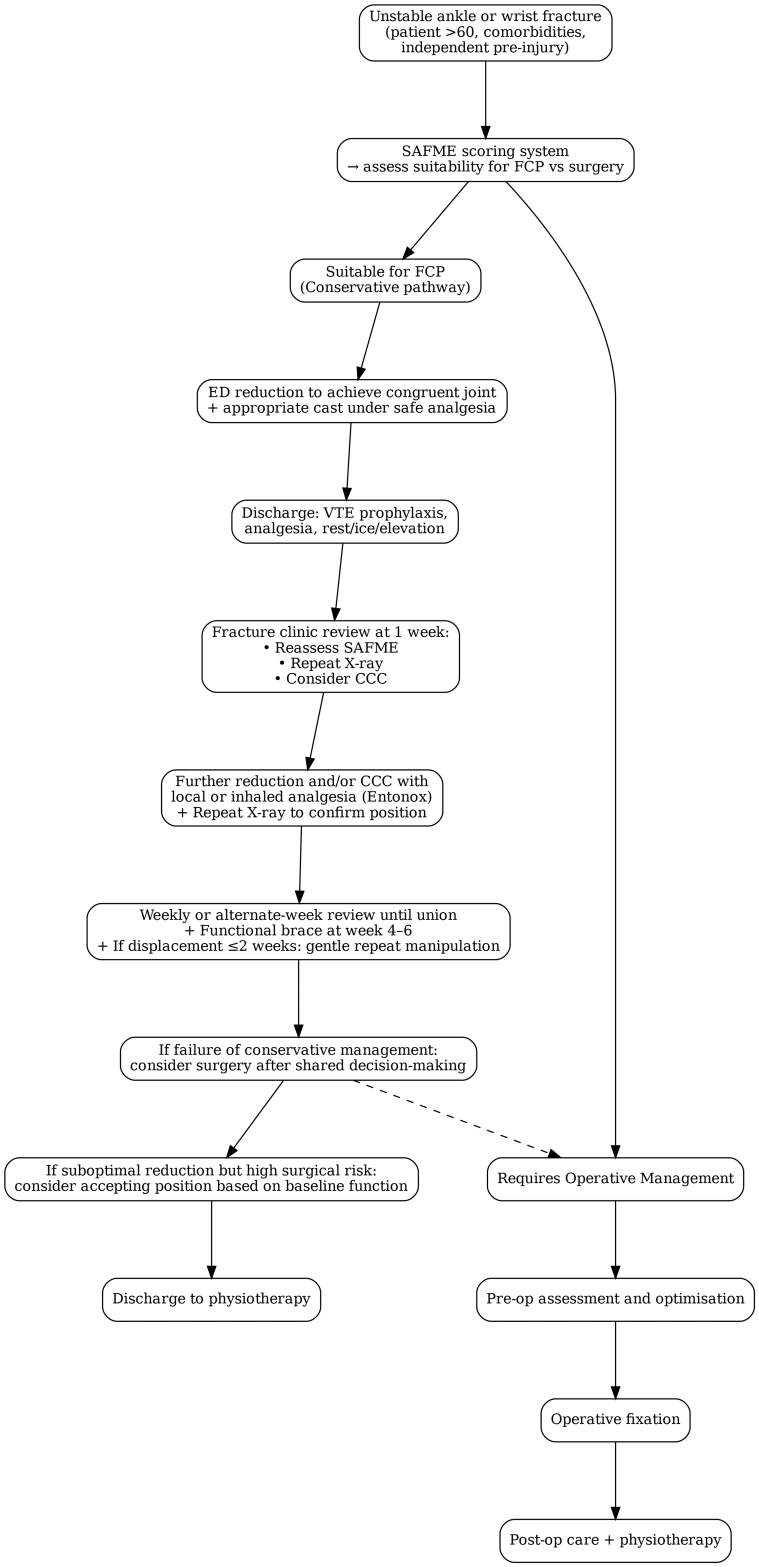
Proposed Fracture Clinic Protocol (FCP) pathway ED - emergency department; VTE - venous thromboembolism; SAFME - Surgical Risk vs Benefit Assessment of Fracture Management in the Elderly; CCC - close contact casting

Following this protocol in the proposed sequence, which includes an initial reduction in ED with further reduction and/or application of CCC a week later, as well as consideration for gentle repeat manipulation until week two post-injury, allows for maximising conservative care. This protocol has been developed to ensure patients identified as having a high SAFME score and therefore more likely to benefit from CCC are appropriately followed up by senior orthopaedic doctors. 

An important consideration that should be included in patient discussion is that there must be a balance struck between the reduction in surgical risks when choosing conservative management, with the known risks of prolonged immobility associated with casting, such as VTE and loss of function. It is for this reason that the FCP proposal includes VTE prophylaxis as well as a proposal of functional bracing at weeks four to six, to help mitigate these risks.

## Discussion

The SAFME scoring tool offers several advantages in healthcare provision

The SAFME scoring tool could revolutionise fracture management in the elderly. Whereas it has been previously considered that operative management produces the most favourable outcomes, there is a lot of research indicating non-operative management may produce similar functional outcomes but is associated with a lot less risk to the patient involved. The SAFME scoring tool uses a multidisciplinary approach to provide an expert-consensus-based scoring tool that can be used to identify patients for whom surgery carries unfavourable risks compared to CCC. The FCP has been constructed to complement the BOAST/ BOFAS recommendations regarding fracture management in the elderly patient. It would reduce the number of inpatient admissions and allow for more utilisation of operating theatres. It also minimises exposure to anaesthesia and post-operative complications whilst enabling the safe continuity of senior-led, patient-centred fracture care.

Challenges and limitations

The scoring bins and interpretation thresholds in the current SAFME scoring tool format have been set arbitrarily and require formal validation. This could be done through receiver operating characteristic curve analysis on retrospective data, before clinical application. Due to its novelty, the scoring bin for a higher probability of benefit from CCC has been set as a high threshold. This means CCC will only be a serious consideration in patients for whom the benefits of CCC greatly outweigh associated surgical risks. 

The decision between surgical and conservative management should be individualised, considering the specific fracture characteristics, the patient's overall health status, functional demands, and personal preferences. As always, there is an ethical need for informed patient consent in considering all management options. The SAFME scoring tool is therefore to be considered an aid to clinical decision making, not a replacement. 

This SAFME scoring tool was designed to incorporate patient demographics considered in orthopaedic and geriatric care in a concise manner. It incorporates aspects of the clinical frailty score, the American Society of Anesthesiologists (ASA) status, and other patient demographic factors deemed necessary to consider when considering management options for bony injuries in elderly adults. By incorporating ASA status, there is also broad incorporation of smoking and alcohol status, as well as significant co-morbidities, all of which affect patient outcomes in fracture management. A limitation of note is that ASA status is used to assess physiological status peri-operatively, and so in this case, it is being used differently, but has been included as it encompasses multiple patient factors which will affect recovery, enabling a more concise and therefore easier to use scoring system. This does leave the scoring system at risk of being an over-simplified representation of a patient's health status.

Specific fracture types may have different thresholds for surgical intervention. The studies in meta-analysis by Javed et al. [21] included seven studies, six of which examined displaced/unstable ankle fractures and one investigated AO-type-B1 fractures with minimal talur shift. The study was unable to perform subgroup analysis based on ankle fracture classification or patient age due to a limited data set and variation in selection criteria of the studies, but did go on to state that the majority of patients included in the study were of Weber B fractures. Therefore, this marks an area of consideration for future studies to consider subgroup analysis of fracture types and their management.

Patients who do not have a recent dual-energy X-ray absorptiometry (DEXA) scan may only have X-ray imaging of injury available. It would be difficult for orthopaedic surgeons to categorise bone health quality into the above categories only based on X-rays. However, the relatively small number of categories (0-3) in the SAFME scoring tool should allow senior orthopaedic doctors to do this using available signs on radiographs, e.g., cortical thinning, endosteal scalloping, and severe comminution with large fragments. This would therefore be judged to be a more subjective score; however, it is still vital to include it as a significant factor that is considered when deciding on surgical vs non-surgical management plans.

There are also foreseeable limitations in the implementation of the SAFME scoring tool. It relies on input from senior orthopaedic doctors, who are already recognised as a limited availability resource. Additionally, application of CCC is a skill that requires training, and so it would need to be considered whether further training was given before using this scoring tool in conjunction with FCP. There is also the vast administrative and institutional support that would be required to expand current fracture clinics to incorporate the additional workload it would entail. 

A possible consideration to test the SAFME scoring tool would be to analyse a dataset of patients who were treated for ankle fractures operatively or using CCC in the form of an observational study. The SAFME scoring tool could be applied retrospectively to determine the scores of patients who were treated operatively or using CCC. This cohort could then be followed up over a significant period of time, which would have to be at least one year, to monitor long-term outcomes. Conducting the study using this method offers multiple advantages. It does leave patients at risk of being treated incorrectly, as the study is not attempting to influence any management plan. However, by collecting data at the time of assessment, it would ensure that all parameters included in the SAFME scoring system are measured at the time of treatment, which may not be possible with retrospective data collection. Another advantage of collecting data at the time of assessment is that it would allow the data collector to notice any other factors that are common considerations when deciding between surgery and CCC that may not already be incorporated into the SAFME scoring tool.

## Conclusions

This report provides a potential blueprint for a system that may not only incorporate cost-saving measures by reducing the need for operative intervention but also provide similar functional scores to surgical management. It also reduces other risks, such as those associated with anaesthesia and post-operative complications, reducing the need for invasive monitoring with lighter forms of analgesia, such as inhaled nitrous oxide and the provision of oral medication. This may prove to be an effective solution in a particular patient cohort, as hopefully guided by the innovation of the SAFME scoring tool. As some medical ailments are often part of a wide spectrum of severity, clinicians should consider the presence of co-morbidities that may have a higher chance of negative impact upon fracture healing. These may be the presence of ulceration in places such as lower limb fractures and cognitive impairment (which may limit compliance with non-weight bearing). The SAFME scoring tool provides a rational basis for choosing between surgical and conservative management using 13 parameters of patient health status. It aims to guide clinicians in their decision-making while encouraging individualised, resource-efficient, patient-centred care. The FCP aims to complement this by ensuring safe, senior-led implementation of conservative care, utilising CCC. Through the adoption of the SAFME scoring tool, the aims would be to reduce surgical complications and improve functional outcomes, whilst aligning treatment strategies with patients' goals of care. However, this scoring tool will require formal validation/ trial in the clinical setting to ensure it is safe for practice and that no patients are treated incorrectly. A consideration to test the efficacy of SAFME would be conducting an observational study of patients who have had surgical or CCC as management of an unstable fracture. This would allow the safe analysis of patients as their treatment plan would not be altered. Retrospective application of SAFME in a real-world setting would allow it to be tested, and its accuracy could be analysed, possibly guiding an updated version of it. As in any case, the options should be discussed, allowing the patient's informed decision, keeping in mind optimal fracture healing and the best possible outcome.
